# Gerstmann Syndrome as the Sole Clinical Manifestation of Ischemic Stroke Secondary to Atrial Fibrillation: A Case Report

**DOI:** 10.7759/cureus.108495

**Published:** 2026-05-08

**Authors:** Paola V Rosales-Verduzco, Ana Laura Rodríguez-Hernández, Ricardo Cid-Puente, Daniel Vidrio-Carrizales

**Affiliations:** 1 Department of Internal Medicine, Hospital Regional Dr. Valentín Gómez Farías Instituto de Seguridad y Servicios Sociales de los Trabajadores del Estado (ISSSTE), Zapopan, MEX; 2 Department of Internal Medicine, Hospital General de Zona No.1 "Dr. Emilio Varela Lujan" Instituto Mexicano del Seguro Social, Zacatecas, MEX

**Keywords:** acalculia, agraphia, atrial fibrillation, gerstmann syndrome, ischemic stroke

## Abstract

Gerstmann syndrome (GS) is a rare neurological disorder characterized by the presence of agraphia, acalculia, right-left disorientation, and finger agnosia. In older adults, the isolated presentation of this syndrome may represent an early sign of an ischemic cerebrovascular event, particularly in patients who have cardioembolic risk factors such as atrial fibrillation (AF). We report the case of a 72-year-old male with a history of hypertension, type 2 diabetes, AF without anticoagulant treatment, and chronic alcohol use who presented with a five-day history of behavioral changes and difficulty communicating. The patient's symptoms were initially attributed to hepatic encephalopathy or possible alcohol poisoning. However, a detailed neurological examination revealed dysgraphia, acalculia, right-left disorientation, and finger agnosia. Cranial magnetic resonance imaging subsequently confirmed a multifocal ischemic lesion in the left temporal and right temporoparietal lobes, establishing a diagnosis of GS secondary to ischemic stroke. This report highlights the importance of early recognition of rare focal neurocognitive syndromes as the sole presentation of cerebrovascular events.

## Introduction

Gerstmann syndrome (GS) is a rare neurological disorder first described by Josef Gerstmann in 1924, characterized by the presence of agraphia, acalculia, right-left disorientation, and finger agnosia [1]. It has been reported that GS often presents as an incomplete tetrad or in association with additional cognitive deficits, including aphasia, alexia, apraxia, and certain perceptual disturbances [2]. These manifestations are classically associated with lesions in the inferior parietal region of the dominant hemisphere, particularly the angular gyrus [3]. Although described in pediatric neurodevelopmental disorders, in adults, it is most often associated with acquired lesions, such as cerebrovascular accidents, neoplasms, trauma, or central nervous system infections [1]. Notably, its isolated presentation in older adults may represent an early sign of an ischemic cerebrovascular event in the parietal territory, particularly in patients with cardioembolic risk factors such as atrial fibrillation (AF).

In this report, we describe the case of a patient who developed GS as the sole neurological manifestation of an ischemic stroke in the left parietal territory. The aim is to highlight the importance of early clinical recognition of this syndrome as a useful topographic marker for stroke diagnosis, especially in patients with cardioembolic risk factors. Furthermore, we underscore the importance of conducting comprehensive neurological evaluations in situations where clinical presentations might be nuanced or uncharacteristic.

## Case presentation

A 72-year-old male, retired physician, with a past medical history significant for hypertension diagnosed 26 years prior (treated with enalapril), type 2 diabetes mellitus of 26 years' duration (managed with preprandial rapid-acting insulin adjusted as needed), and AF without anticoagulant therapy (managed solely with propafenone). The patient also reported chronic alcohol use since childhood, with an estimated daily intake of 500 mL of tequila. He denied smoking or illicit drug use.

The patient's symptoms began five days prior to hospital admission while at home. Day 0: symptom onset with behavioral changes, difficulty communicating, lack of eye contact, and sudden inattention, with a fluctuating course. At that time, precipitating events such as head trauma were ruled out. Family members attributed the symptoms to possible alcohol intoxication, given the history of chronic use. Days 1-4: progressive psychomotor agitation and worsening language disturbances developed with more evident aphasia. Despite this, medical attention was not sought. Day 5: Hospital admission, due to lack of improvement and progressive deterioration, the family decided to seek emergency care.

Upon admission to the emergency department, vital signs were blood pressure of 139/74 mmHg, heart rate of 95 bpm, respiratory rate of 18 breaths per minute, and oxygen saturation of 95% on room air. History was obtained indirectly through the patient's daughter due to impaired communication.

The patient was subsequently admitted to the internal medicine service for further evaluation. A detailed neurological examination revealed the following: (1) mental status and cognition: temporospatial disorientation, decreased attention and concentration, mildly impaired short-term memory, preserved long-term memory; (2) language: disorganized spontaneous speech, reduced fluency, phonemic paraphasia, semantic deficits, partially preserved naming, intact repetition, and presence of neologisms; (3) cranial nerves: no abnormalities detected; (4) motor: normal tone and strength (5/5 in all extremities, proximal and distal, according to the Medical Research Council (MRC) scale), without atrophy; (5) reflexes: deep tendon reflexes were ++/++++ in biceps, triceps, brachioradialis, patellar, and Achilles tendons; (6) coordination was intact, without dysmetria or dysdiadochokinesia; (7) sensory: exteroceptive sensation was intact; however, proprioceptive assessment revealed finger agnosia; (8) coordination: no dysmetria or dysdiadochokinesia; and (8) additional findings: acalculia, dysgraphia, and right-left disorientation.

**Figure 1:**
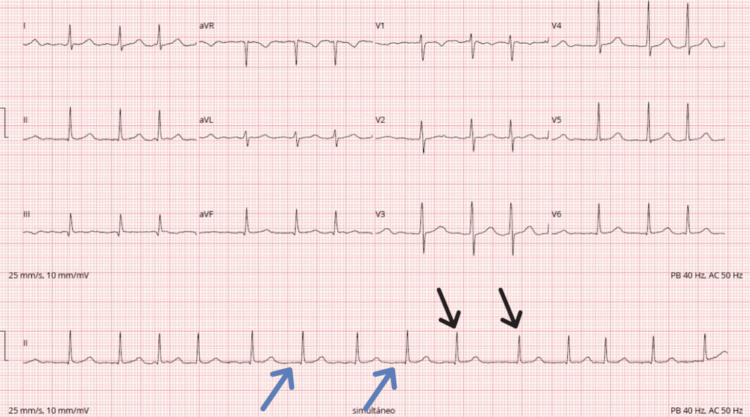
12-lead electrocardiogram The electrocardiogram shows irregular R-R intervals (black arrows) and absence of P waves (blue arrows), consistent with atrial fibrillation.

**Figure 2:**
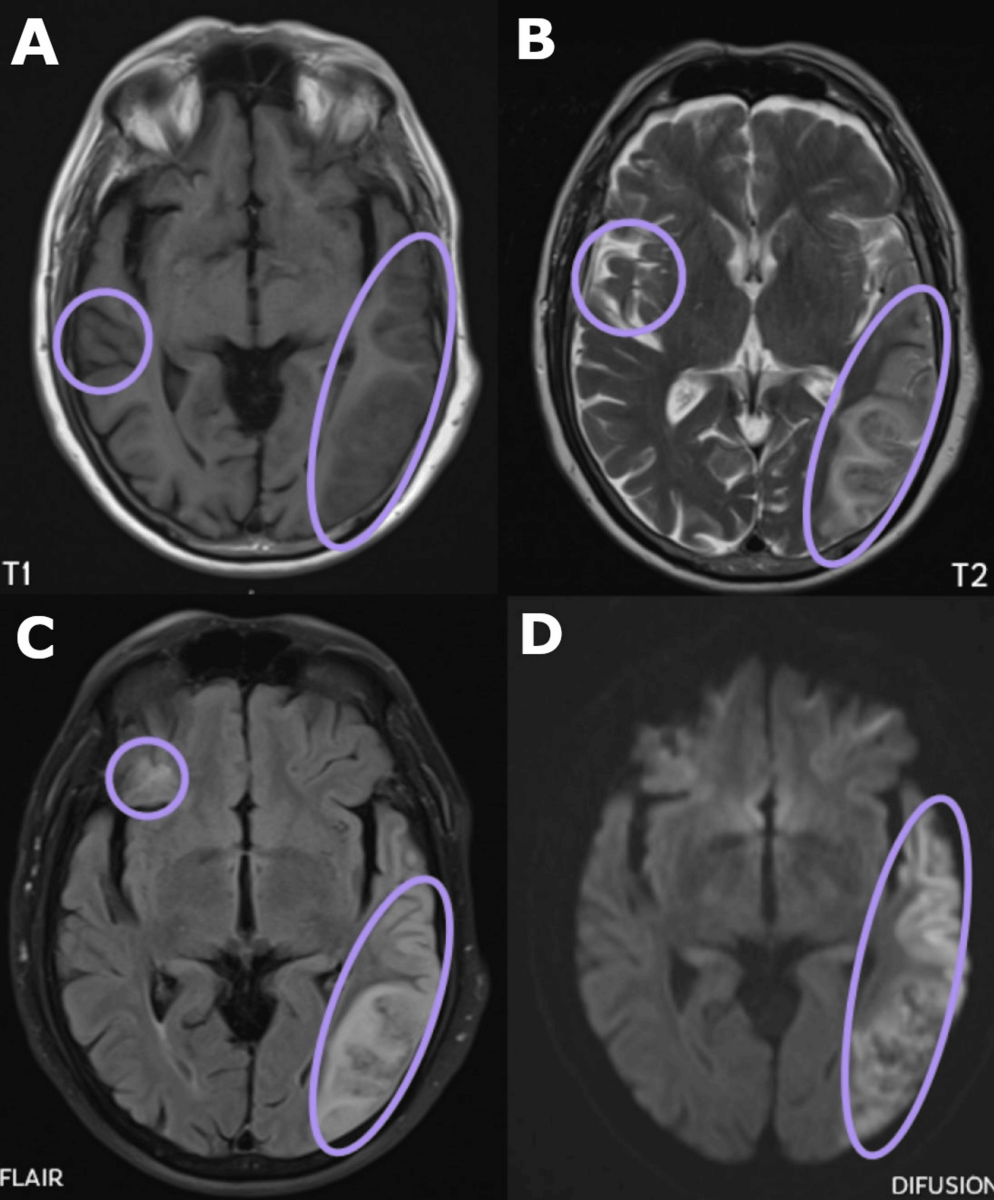
Simple cranial magnetic resonance imaging Panel A (T1 sequence) demonstrates hypointense regions (outlined by purple circles) within the left temporal and right temporoparietal lobes. Panels B (T2 sequence), C (fluid-attenuated inversion recovery (FLAIR) sequence), and D (diffusion sequence) reveal hyperintense areas (highlighted by a purple circles) accompanied by sulcal effacement in the right temporoparietal and left temporal lobes, which are indicative of cerebral ischemia

The NIH Stroke Scale (NIHSS) score was 5, corresponding to a moderate neurological deficit. Laboratory studies were within normal limits (Table 1). The electrocardiogram showed findings consistent with AF (Figure 1). Due to the neurological deficit presented by the patient, cranial magnetic resonance imaging was performed, revealing hypointense regions within the left temporal and right temporoparietal lobes suggestive of cerebral ischemia (Figure 2).

**Table 1:** Laboratory results upon admission to the emergency department

Laboratory tests	Admission	Reference range
Serum creatinine (mg/dL)	1.10	0.6-1.2
Urea (mg/dL)	40.0	15.0-40.0
Aspartate aminotransferase (UI/L)	11.0	17.0-59.0
Alanine aminotransferase (UI/L)	24.0	0.0-50.0
Conjugated bilirubin (mg/dL)	0.56	0.0-0.3
Unconjugated bilirubin (mg/dL)	1.14	0.0-1.1
Lactate dehydrogenase (U/L)	177.0	120.0-246.0
Alkaline phosphatase (U/L)	92.0	38.0-126.0
Cholesterol (mg/dL)	154	< 200
Triglycerides (mg/dL)	121	< 150
Low density lipoprotein (mg/dL)	88	< 100
Glucose (mg/dL)	165	< 130
Glycosylated hemoglobin (HbA1c) (%)	7	< 5.7
Calcium (mg/dL)	9.3	8.5-10.5
Phosphorus (mg/dL)	3.6	2.5-4.5
Sodium (mmol/L)	136	135-145
Magnesium (mg/dL)	1.9	1.6-2.6
Potassium (mmol/L)	3.8	3.5-5.0
Hemoglobin (g/dL)	13.6	14.0-16.0
White blood count (1,000/uL)	7.73	4.5-11.0
Platelets (1,000/uL)	221	150.0-450.0
Ammonium (ug/dL)	23	15-45
Fibrinogen (mg/dL)	577	200-400
D-dimer (mg/dL)	370	< 500
Thyroid-stimulating hormone (mUI/L)	3.2	0.4-4.0
Toxicology ethanol test (mg/dL)	0	0

Based on clinical and imaging findings, a diagnosis of GS secondary to ischemic stroke was established. A transthoracic echocardiogram was performed, revealing no structural abnormalities or intracavitary thrombi. The patient was started on supportive care, anticoagulation, and neurorehabilitation. Clinical evolution was favorable, and the patient was discharged with outpatient follow-up and rehabilitation referral.

Given a CHA₂DS₂-VA score of 4, the patient was discharged on apixaban of 5 mg twice daily, propafenone of 150 mg, and atorvastatin of 80 mg daily. Dual antiplatelet therapy was not initiated, considering a NIHSS score of 5 and a dual antiplatelet therapy (DAPT) score of 0; therefore, only acetylsalicylic acid 150 mg daily was prescribed. Neuropsychology consultation was requested for language rehabilitation. After five days of hospitalization, the patient was discharged with clinical improvement.

## Discussion

This case documents a cerebrovascular event with an unusual clinical manifestation associated with cardiovascular etiology. The patient initially presented with behavioral changes, aphasia, and inattention of acute onset, with a fluctuating course over five days. This led to consideration of differential diagnoses, such as hepatic encephalopathy or alcohol intoxication, as the initial presentation lacked clear localizing value and was not explained by laboratory findings.

Subsequently, the patient developed more specific clinical signs, including acalculia, agraphia, right-left disorientation, and finger agnosia, which constitute the classical components of GS [4-7].

Given that this syndrome is most commonly of vascular origin, neuroimaging with magnetic resonance imaging was performed, confirming the etiology. Cortico-subcortical lesions were identified in the right parietal region and left parieto-occipital subcortical areas, involving the angular and supramarginal gyri, findings consistent with those described in the literature [8].

Although this condition is typically associated with lesions in the dominant parietal lobe, particularly the angular gyrus, this case demonstrated subacute lesions in multiple locations, suggesting a cardioembolic origin in a patient with AF, even without a fully evident clinical correlation [5,8].

It is important to note that, although specific functions are attributed to individual cortical regions, it is not uncommon to observe impairment in multiple cognitive domains, or even motor or sensory deficits, associated with focal neurological lesions. This may be explained by functional disconnecting between cortical areas involved in cognitive processing, as described in neuroanatomical and functional studies of Gerstmann syndrome [6].

While the syndrome classically localizes to the dominant inferior parietal lobule, specific features often map to distinct sub-regions: agraphia (dominant parietal lobe, often involving the superior parietal lobule or supramarginal gyrus), acalculia (angular gyrus), and right-left disorientation (parietal association cortex) (Table 2). Pure cortical lesions of the angular gyrus rarely produce all four symptoms simultaneously. Modern neuroimaging reveals that the full syndrome is frequently the result of subcortical white matter damage, often from multifocal ischemic events [2].

**Table 2:** Key diagnostic features of Gerstmann syndrome The presence of this classic tetrad strongly suggests a lesion in the dominant inferior parietal lobule, specifically involving the angular gyrus.

Clinical Feature	Description	Example of Clinical Testing
Agraphia (or Dysgraphia)	An acquired inability to write or spell. This is a central language defect, not a motor deficit.	Ask the patient to write a spontaneous sentence, write a dictation, or copy written text
Acalculia (or Dyscalculia)	An acquired inability to perform simple mathematical calculations or understand mathematical principles.	Ask the patient to perform basic arithmetic
Finger Agnosia	The inability to recognize, identify, differentiate, or name the fingers on one's own hands, the examiner's hands, or a drawing.	Ask the patient to name a specific finger the examiner touches or ask them to show their index finger
Left-Right Disorientation	An inability to distinguish right from left on oneself, on the examiner, or in the environment.	Ask the patient to execute crossed commands, such as "Touch your right ear with your left index finger."

In this case, the presence of multiple lesions posed a diagnostic challenge, requiring consideration of other possible etiologies for a subacute process described in the literature, including infectious, neoplastic, demyelinating, autoimmune conditions, intoxications, or decompensation of chronic diseases [8].

It is important to emphasize that, in the initial evaluation of patients with suspected cerebrovascular disease, a comprehensive neurological examination and detailed history-taking should be performed early, especially in emergency settings. However, in many cases, the initial evaluation is limited to standardized scales, such as the NIHSS, which may hinder the identification of more specific neurocognitive deficits [5].

These limitations, along with difficulties in patient interaction or communication and the lack of active assessment for neurocognitive impairment, may represent diagnostic obstacles and even contribute to epidemiological bias.

Therefore, more detailed neuropsychological evaluations are essential to establish an accurate diagnosis and provide individualized treatment, including cognitive rehabilitation strategies and pharmacological therapy when necessary [9].

## Conclusions

GS represents a rare neurological manifestation that retains significant semiological value as a topographic marker of lesions in the dominant parietal lobe. This case demonstrates that this constellation of neurocognitive alterations may constitute the primary clinical manifestation of an ischemic cerebrovascular event, even in the absence of evident motor or sensory deficits, potentially complicating its recognition during initial evaluation. This report highlights the importance of conducting a detailed neurological examination in patients presenting with acute cognitive or language disturbances, particularly those with cardiovascular risk factors. Identification of characteristic findings, such as agraphia, acalculia, right-left disorientation, and finger agnosia, allows for accurate anatomical localization and improved clinicoradiological correlation. Early recognition of rare focal neurocognitive syndromes may contribute to the timely diagnosis of atypical cerebrovascular events within more complex or multifocal cerebrovascular presentations, enabling the prompt initiation of targeted therapeutic and rehabilitation strategies, with the potential to improve functional outcomes.
